# Proteomic profiling of tumour tissue‐derived extracellular vesicles in colon cancer

**DOI:** 10.1002/jex2.127

**Published:** 2024-02-06

**Authors:** Aleksander Cvjetkovic, Nasibeh Karimi, Rossella Crescitelli, Annika Thorsell, Helena Taflin, Cecilia Lässer, Jan Lötvall

**Affiliations:** ^1^ Krefting Research Centre, Institute of Medicine at Sahlgrenska Academy University of Gothenburg Gothenburg Sweden; ^2^ Sahlgrenska Center for Cancer Research and Wallenberg Centre for Molecular and Translational Medicine, Department of Surgery, Institute of Clinical Sciences, Sahlgrenska Academy University of Gothenburg Gothenburg Sweden; ^3^ Department of Surgery Sahlgrenska University Hospital Gothenburg Sweden; ^4^ Proteomics Core Facility, Sahlgrenska Academy University of Gothenburg Gothenburg Sweden; ^5^ Transplant Institute at Sahlgrenska University Hospital, Institute of Clinical Sciences Sahlgrenska Academy at University of Gothenburg Gothenburg Sweden

**Keywords:** biomarkers, colorectal cancer, extracellular vesicles, proteomics, tumour microenvironment

## Abstract

Colon cancer is one of the most commonly occurring tumours among both women and men, and over the past decades the incidence has been on the rise. As such, the need for biomarker identification as well as an understanding of the underlying disease mechanism has never been greater. Extracellular vesicles are integral mediators of cell‐to‐cell communication and offer a unique opportunity to study the machinery that drives disease progression, and they also function as vectors for potential biomarkers. Tumour tissue and healthy mucosal tissue from the colons of ten patients were used to isolate tissue‐resident EVs that were subsequently subjected to global quantitative proteomic analysis through LC‐MS/MS. In total, more than 2000 proteins were identified, with most of the common EV markers being among them. Bioinformatics revealed a clear underrepresentation of proteins involved in energy production and cellular adhesion in tumour EVs, while proteins involved in protein biosynthesis were overrepresented. Additionally, 53 membrane proteins were found to be significantly upregulated in tumour EVs. Among them were several proteins with enzymatic functions that degrade the extracellular matrix, and three of these, Fibroblast activating factor (FAP), Cell surface hyaluronidase (CEMIP2), as well as Ephrin receptor B3 (EPHB3), were validated and found to be consistent with the global quantitative results. These stark differences in the proteomes between healthy and cancerous tissue emphasise the importance of the interstitial vesicle secretome as a major player of disease development.

## 1 INTRODUCTION

Colorectal cancer is one of the most frequently occurring types of cancer and has similar prevalence in both women and men, and it is expected to increase in incidence (wcrf.org, August 2021). Thus, the need for a deeper understanding of the disease mechanisms that drive malignancy as well as biomarkers for disease detection are ever increasing.

Extracellular vesicles (EVs) are nano‐sized membrane‐bound vesicles secreted by cells (Yáñez‐Mó et al., 2015), and in recent years they have been shown to play a major part in cancer biology (Ciardiello et al., 2016; Costa‐Silva et al., 2015; Di Vizio et al., 2012; Jung et al., 2009; Luga et al., 2012; Skog et al., 2008). Recognised as a basic means of cell‐to‐cell communication, EVs have been shown to play a myriad of different roles in promoting tumour progression as well as in facilitating its regression (Al‐Nedawi et al., 2008; Bigagli et al., 2016; Chiba et al., 2016; Hood et al., 2011; Taylor et al., 2003). In addition to their lipid bilayer shell, they have been shown to carry protein cargo as well as genetic material in the form of DNA and different RNA species (Haraszti et al., 2016; Valadi et al., 2007; Yáñez‐Mó et al., 2015). After being released, these vesicles can be taken up and potentially induce a response in recipient cells (Al‐Nedawi et al., 2008; Taylor et al., 2003; Valadi et al., 2007).

The fact that EVs carry components of their parent cells and thus reflect aspects of those cells’ phenotypes, and their ability to spread to other parts of the body such as the circulating blood, makes EVs excellent candidates for biomarkers (Rajagopal & Harikumar, 2018). Furthermore, EVs have been shown to be packed with specifically selected components that can induce activities in recipient cells, making them interesting from a treatment perspective as well (Zitvogel et al., 1998). In essence, EVs can be viewed as a mobile extension of an otherwise spatially confined cell.

Although several studies have investigated the role of EVs in cancer and their potential as biomarkers, only a few studies have used EVs derived directly from the diseased tissue, and most have instead used either cancer cell lines as model systems or have gone directly to body fluid‐derived EVs (Buenafe et al., 2022; Crescitelli et al., 2020; Hoshino et al., 2020; Jang et al., 2019; Jeurissen et al., 2017; Lane et al., 2018; Pane et al., 2022; Shiromizu et al., 2017). The main benefit of investigating tissue‐derived EVs is that it is in the tumour microenvironment that the cancer secretome is at its peak concentration. Thus, a readout is less confounded by non‐tumour entities as would be the case in the circulation. Furthermore, tissue‐derived EVs would be more relevant to the disease than their cell line counterparts. We recently showed that melanoma tissue contains a subpopulation of EVs that are enriched in mitochondrial inner membrane proteins, and this subpopulation could be further identified in the circulation in melanoma patients (Crescitelli et al., 2020; Jang et al., 2019). However, little is known about colon cancer EVs in the tumour tissue and in circulation. We therefore isolated EVs directly from the tissue interstitium of colon cancer tumours and subsequently analysed the protein cargo by global quantitative mass spectrometry in order to profile the vesicular secretome of the tumour. As controls, macroscopically normal colonic mucosa was sampled, hereafter referred to simply as mucosa.

The good coverage of proteins from both tumour EVs and mucosal EVs revealed a clear distinction between the two in terms of cargo. Dysregulation of proteins tied to several biological pathways—such as those related to protein production, cellular adhesion and oxidative respiration—suggests a unique tumour‐associated secretome that carries clues as to the mechanism of disease progression. Furthermore, several consistently dysregulated proteins stand out as potential biomarker candidates, such as FAP and CEMIP2 among others. The differences in protein expression between healthy and diseased tissue as well as the most highly dysregulated proteins together illustrate the value of an approach targeting the vesicular secretome at its source in the tumour microenvironment of the patient.

## 2 MATERIALS AND METHODS

### 2.1 Tissue processing

Fresh tissue specimens were acquired during tumour resection surgery. Both tumour tissue and macroscopically normal appearing mucosa located 10 cm from the tumour were retrieved and micro dissected in such a manner as to avoid muscularis mucosae and submucosa. In total, samples were retrieved from 10 consenting colorectal cancer patients undergoing surgery, resulting in a total of 20 tissue specimens. Patient demographics are given in Table 1. The patients had given their informed consent for data to be collected for study purposes and is approved by the Regional Ethical Review Board in Gothenburg (DNR 118‐15).

**TABLE 1:** Patient demographics.

Patient	Sex	Age	TNM‐stage	Sample used for
A	Female	75	pT4N1a	TEM
B	Male	71	pT3bN1b	TEM
C	Male	68	pT3b1N0	TEM
1	Male	74	pT2N0	LC‐MS/MS
2	Female	86	pT3bN2a	LC‐MS/MS
3	Female	78	pT3N0	LC‐MS/MS
4	Female	56	pT4aN1b	LC‐MS/MS, ExoView, NTA
5	Female	75	pT3bN0	LC‐MS/MS, WB, NTA
6	Male	83	T3bN0	LC‐MS/MS, WB
7	Female	82	T2N0	LC‐MS/MS
8	Female	61	T3cN2a	LC‐MS/MS, WB
9	Female	74	T3dN0	LC‐MS/MS, WB, NTA
10	Female	87	T4aN0	LC‐MS/MS

Patient demographics and which analysis the samples were used for. Patients denoted alphabetically were not included in the proteomics, while patients denoted by numbers were included. Tandem liquid chromatography/mass spectrometry (LC‐MS/MS), Western blots (WB), Transmission electron microscopy (TEM).

**FIGURE 1:**
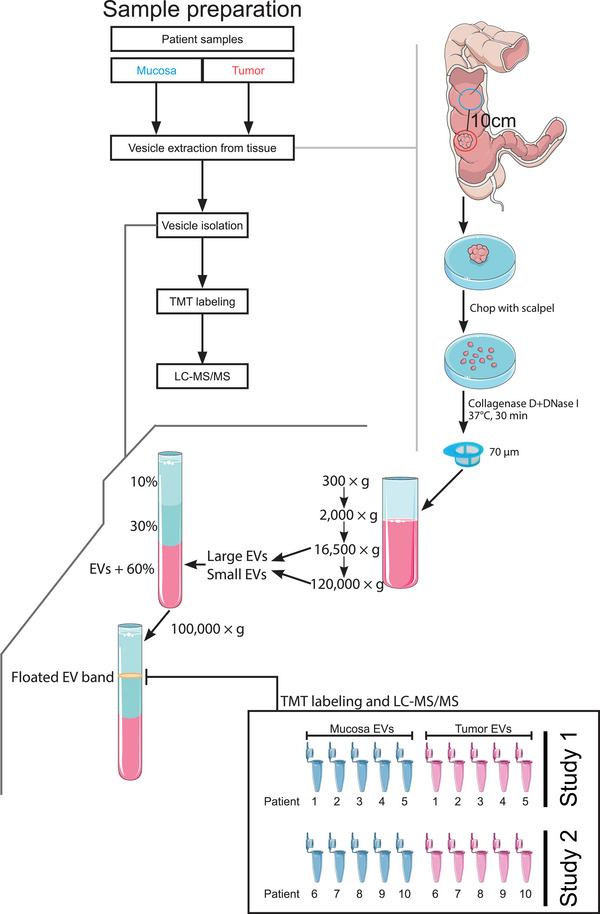
Schematic overview of the experimental workflow. Tissue samples were collected from colorectal cancer patients during surgery. Each patient yielded one tumour tissue sample and one non‐tumour ‘mucosa’ tissue sample to serve as the control, and these were immediately subjected to vesicle extraction after excision. The extraction was performed by a combination of both mechanical and enzymatic means. By cutting the tissue into smaller pieces, EVs were allowed to more readily diffuse into the medium. Collagenase D digestion further aided in this by degrading the extracellular matrix, while DNase 1 prevented the formation of a sticky pellet further downstream in the isolation protocol (Crescitelli et al., 2020). Following the release of EVs into the medium, filtration and a sequential centrifugation protocol were applied to eliminate large tissue pieces, free cells, and cell debris. Lastly, EVs were isolated through an ultracentrifugation protocol with a density cushion flotation as the last step, where buoyant EVs could be collected at the intersection of 1.078 and 1.175 g/mL Optiprep. Protein extraction, digestion, and TMT labelling preceded the final step of mass spectrometry analysis.

A similar approach to that of Crescitelli et al. was employed for the extraction of EVs from the tissue interstitium (Crescitelli et al., 2021, 2020). Tissue pieces were weighed and then divided into approximately 0.2 g pieces, each of which was placed in a well of a 6‐well plate containing 2 mL plain, unsupplemented RPMI medium (Sigma Aldrich). The pieces were then chopped into approximately 1 mm^3^ fragments and DNase 1 (Roche; cat. no. 11284932001) and Collagenase D (Roche; cat. no. 11088858001) were added to a final concentration of 40 U/mL and 2 mg/mL, respectively (Figure 1). The plates were then incubated under gentle agitation at 37°C for 30 min. After incubation, the samples were passively passed through a 70 µm filter and the wells were further rinsed with 1 mL of fresh RPMI medium that was also passed through the filter. The resulting filtered liquid, now void of visible tissue pieces, was used for EV isolation.

### 2.2 EV isolation

Extracellular vesicles from tissues were isolated as previously described (Crescitelli et al., 2021). Briefly, the tissue‐conditioned fluid was centrifuged at 300 × *g* for 10 min to remove any potentially contaminating cells from the tissue (Figure 1). The supernatant was then centrifuged again at 2000 × *g* for 20 min to remove cell debris and very large vesicles such as apoptotic bodies. Larger EVs were then pelleted by ultracentrifugation at 16,500 × *g*_avg_ for 6 min (TLA 100.3, k‐factor: 404.5, Beckman Coulter). The vesicle pellet was resuspended in PBS and the supernatant was ultracentrifuged at 120,000 × *g*_avg_ for 65 min (TLA 100.3, k‐factor: 55.5, Beckman coulter) to pellet smaller EVs. This pellet was also resuspended in PBS. The two pellets enriched for large and small EVs were pooled, and the EVs were further purified by a bottom‐loaded iodixanol density cushion (Optiprep density gradient, Sigma Aldrich). Briefly, the pooled EVs were bottom‐loaded by mixing 1 mL sample with 3 mL of 60% Optiprep, which was placed at the bottom of an ultracentrifuge tube. On top of this, 4 mL of 30% Optiprep and 4 mL of 10% Optiprep was carefully layered. The samples were then centrifuged at 100,000 × *g*_avg_ for 2 h (SW 41 Ti, k‐factor: 265.1, Beckman Coulter). After centrifugation, the visible band containing the purified EVs was collected from the 10%/30% interface with a pipette. The protein concentration was estimated with the Qubit assay system as per the manufacturer's instructions (Thermo Fischer Scientific).

### 2.3 Western blot

Patient sample protein extracts were generated by lysis using RIPA buffer and then separated by SDS‐PAGE on a precast polyacrylamide gel (Mini‐Protean TGX 4–12% gel, Bio‐Rad Laboratories, Hercules, CA, USA). Transfer onto PVDF membranes was performed using the Turbo‐Transfer system (Bio‐Rad), and the membranes were blocked for 2 h in blocking buffer (TBST containing 5% non‐fat dry milk). Membranes were then probed with primary antibodies against Flotillin‐1 (1:1000 dilution, clone EPR6041, Abcam), CD9 (dilution 1:1000, clone MM2/57, Millipore), CD81 (dilution 1:1000, clone M38, Abcam), CD63 (dilution 1:1000, anti‐CD63, clone H5C6, BD Biosciences), ADAM10 (dilution 1:500, anti‐ADAM10, clone 163003, R&D systems, Minneapolis, MN), Mitofilin (dilution 1:500, Invitrogen), FAP (anti‐FAP, AF3716, R&D systems), EPHB3 (anti‐EPHB3, HPA008184, Merck) and CEMIP2 (anti‐CMIP2, HPA044889, Merck) diluted 1000 times (if not stated otherwise) in blocking buffer overnight at 4°C. Membranes were washed three times in TBST and then probed with horseradish peroxidase conjugated secondary antibodies (donkey anti‐rabbit IgG HRP‐linked F(ab′)2 fragment (1:10,000 dilution; NA9340V and sheep anti mouse IgG HRP‐linked F(ab)2 fragment (1:10,000 dilutions; NA9310V, GE Healthcare) diluted 10,000 times in blocking buffer for 1 h at room temperature. Membranes were then washed an additional three to four times in TBST before addition of SuperSignal West Femto maximum sensitivity substrate (Thermo Fisher Scientific) and analyzed with a ChemiDoc Imaging System (Bio‐Rad).

### 2.4 Electron microscopy

Extracellular vesicle analysis by negative staining was performed as previously described (Crescitelli et al., 2020). Briefly, 10 µg of EVs was placed onto glow discharged 200‐mesh, formvar/carbon‐coated copper grids (Electron Microscopy Sciences, Hatfield, PA). After two washes in H_2_O, EVs were fixed in 2.5% glutaraldehyde. After two further washes in water, the samples were contrasted with 2% uranyl acetate for 1.5 min. Images were obtained using a digitised LEO 912AB Omega electron microscope (Carl Zeiss SMT, Mainz, Germany) at 120 kV equipped with a Veleta CCD camera (Olympus‐SiS, Münster, Germany).

### 2.5 Nanoparticle tracking analysis

Particle concentration was determined for both non‐tumour and tumour samples from three patients using aliquots of the cushioned isolates (see EV isolation) and diluted 1000 or 10,000 times in 0.2 µM filtered PBS. Measurements were performed using a ZetaView PMX 110 instrument (Particle Metrix), and data were analysed using the ZetaView analysis software version 8.2.30.1. The measurement was carried out at all 11 different positions, the video quality was set to medium, and the camera sensitivity was set to 80. with a minimum size of 5, a maximum size of 5000, and a minimum brightness of 20 were used.

### 2.6 ExoView analysis

Surface epitopes of EVs were evaluated using the ExoView Plasma Tetraspanin kit on an ExoView R100 (NanoView Biosciences, Boston) according to the manufacturer's instructions.

In order to load equal amounts of EVs in the assay, the concentration of EVs was measured by nanoparticle tracking analysis as described above, and a total of 1 × 10^8^ particles was taken from the samples and were diluted 1:1 by volume using incubation solution. A total volume of 35 µL of each sample was loaded onto the chips and incubated at room temperature overnight. The chips were then washed three times with incubation buffer followed by incubation with fluorescently labelled antibodies against CD81, CD63 and CD9 diluted in incubation buffer supplemented with 1% BSA for 1 h at room temperature. The chips were further washed with washing buffer three times followed by washing with rinse buffer after which they were scanned using the ExoView apparatus. Analysis was performed using NanoViewer software version 2.6.0 (NanoView Biosciences).

### 2.7 Protein digestion, peptide labelling and fractionation

In total, 10 healthy mucosa and 10 tumour tissue samples from 10 patients were used for the proteomic analysis (Figure 1). The paired samples from patient 1–5 and patient 6–11 were analysed in two separate and independent TMT‐studies, study 1 (February 2018) and the confirmative Study 2 (October 2018), respectively. A representative and identical reference pool were analysed in both study. The reference pool was made up of equal parts from all 10 samples in study 1.

The EV samples were lysed by addition of SDS to a final concentration of 2%. The samples (50 µg) were digested with trypsin using the filter‐aided sample preparation method (Wisniewski et al., 2009). Briefly, samples were reduced with 100 mM dithiothreitol at 60°C for 30 min, transferred to 30 kDa MWCO Pall Nanosep centrifugation filters (Sigma‐Aldrich) and washed repeatedly with 8 M urea and once with digestion buffer (1% sodium deoxycholate in 50 mM TEAB) prior to alkylation with 10 mM methyl methanethiosulfonate in digestion buffer for 30 min. Digestion was performed in digestion buffer by addition of 0.5 µg Pierce MS‐grade trypsin (Thermo Fisher Scientific) at 37°C and incubated overnight. An additional portion of trypsin was added and incubated for another 2 h. Peptides were collected by centrifugation.

Digested peptides were labelled using TMT 11‐plex isobaric mass tagging reagents (Thermo Scientific) according to the manufacturer's instructions, and sodium deoxycholate was removed by acidification with 10% trifluoroacetic acid solution. The combined samples were pre‐fractionated with basic reversed‐phase chromatography using a Dionex Ultimate 3000 UPLC system (Thermo Fischer Scientific). Peptide separations were performed using a reversed‐phase XBridge BEH C18 column (3.5 µm, 3.0 mm × 150 mm, Waters Corporation) and a linear gradient from 3% to 40% solvent B over 17 min followed by an increase to 100% B over 5 min. Solvent A was 10 mM ammonium formate buffer at pH 10.00, and solvent B was 90% acetonitrile and 10% 10 mM ammonium formate at pH 10.00. The fractions were concatenated into 20 fractions, dried, and reconstituted in 15 uL of 3% acetonitrile and 0.2% formic acid.

### 2.8 Nanoflow liquid chromatography/mass spectrometry analysis and database search

Each fraction (2 uL Study1, 3 uL study 2) was analysed on an Orbitrap Fusion Tribrid mass spectrometer interfaced with an Easy‐nLC 1200 nanoflow liquid chromatography system (Thermo Fisher Scientific). Peptides were trapped on an Acclaim Pepmap 100 C18 trap column (100 µm × 2 cm, particle size 5 µm, Thermo Fischer Scientific) and separated on an in‐house packed analytical column (particle size 3 µm, Reprosil‐Pur C18, Dr. Maisch, 75 µm × 300 mm (study1) or 360 mm (study 2)) using a linear gradient from 6% to 32% B over 75 min followed by an increase to 50% B over 5 min and then 100% B for 5 min at a flow rate of 300 nL/min. Solvent A was 0.2% formic acid and solvent B was 80% acetonitrile in 0.2% formic acid. Precursor ion mass spectra were acquired at 120,000 resolution, and MS2analysis was performed in a data‐dependent mode where the most intense doubly or multiply charged precursor ions were isolated in the quadrupole with a 0.7 m/z isolation window and dynamic exclusion within 10 ppm for 45s (study 1) or 60 s (study 2). The isolated precursors were fragmented by collision induced dissociation (CID) at 30% collision energy for 3 s (‘top speed’ setting) and detected in the ion trap, followed by multinotch (simultaneous) isolation of the top 7 (study 1) or 10 (study 2) MS2 fragment ions were fragmented (MS3) by higher‐energy collision dissociation (HCD) at 65% collision energy and detection in the Orbitrap at 50 000 resolution.

### 2.9 Database search and quantification

The data files for each study were merged for identification and relative quantification using Proteome Discoverer version 2.2 (Thermo Fisher Scientific). The search was against Homo Sapiens Swissprot Database (Nov 2017) using Mascot 2.5 (Matrix Science) as the search engine with a precursor mass tolerance of 5 ppm and a fragment mass tolerance of 0.6 Da. Tryptic peptides were accepted with zero missed cleavages, variable modifications of methionine oxidation, fixed cysteine alkylation, and TMT‐labelled modifications of the N‐terminus and lysines. Percolator was used for PSM validation with the strict FDR threshold of 1%. TMT reporter ions were identified with 3 mmu mass tolerance in the MS3 HCD spectra, and the TMT reporter abundance values for each sample were normalized on the total peptide amount. Only the quantitative results for the unique peptide sequences with the minimum synchronous precursor selection (SPS) match % of 65 and the average S/N above 10 were taken into account for the protein quantification. The reference samples were used as denominator and for calculation of the abundance ratios in each set. The quantified proteins were filtered at 1% FDR at protein level and grouped by sharing the same sequences to minimise redundancy.

### 2.10 Enrichment analysis

Gene ontology analysis was performed through the web‐based platform g.Profiler (version e107_eg54_p17_bf42210). The web‐based tool NetworkAnalyst (https://www.networkanalyst.ca, 2020‐01‐12) was used to generate a network based on the 2271 proteins identified in both studies by taking data from the KEGG database (https://www.genome.jp/kegg/) (Zhou et al., 2019).

### 2.11 Principal component analysis

A principal component analysis (PCA) was performed using the Qlucore Omics Explorer (Qlucore, Lund, Sweden). The three components that best explained the variability in the data were plotted for the 2271 overlapping proteins between the two sets.

### 2.12 Statistics and calculations

Sample abundance ratios were generated by dividing individual abundances to the reference pool. To identify significantly changed proteins, a paired t‐test was applied to Log2 transformed protein ratios where a p‐value of 0.05 or lower was considered as significant. Individual paired fold changes were calculated by subtracting the Log2 transformed abundance ratios of tumour sample with that of mucosa sample. Throughout this work, we refer to relative differences in quantified protein levels as either upregulated or downregulated for proteins with higher relative abundances in tumour‐derived EVs or mucosa‐derived EVs respectively.

## 3 RESULTS

### 3.1 EVs are isolated directly from colon tumour tissue

A scheme for the extraction and isolation of EVs from the tissue microenvironment was adapted from recent papers using melanoma tumour tissue (Crescitelli et al., 2021, 2020; Jang et al., 2019) and is illustrated in Figure 1. Briefly, a combination of both mechanical and enzymatic treatments was used to extract EVs from the tissue interstitium into the immersion medium. Larger structures such as tissue debris were filtered out, after which the flow‐through was subjected to a regime of isolation and purification including differential ultracentrifugation and density flotation to attain a final isolate to be used for proteomics analysis.

**FIGURE 2:**
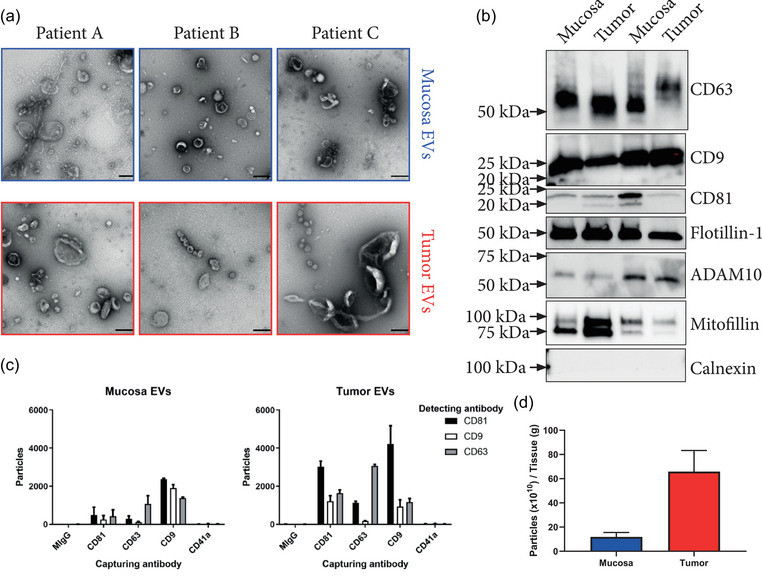
Analysis of tissue Isolates. (a) Electron micrographs showing the vesicle isolates of three patients from both mucosa and tumour tissue. These three patient samples were not included in the proteomics analysis. Scale bars = 200 nm. (b) Tumour and mucosa isolates of two patients were probed for CD9, CD63, CD81, Flotillin 1, ADAM10, Mitofilin and Calnexin by western blot. (c) ExoView analysis of EVs captured by immobilised antibodies against CD9, CD63, CD81 and CD41a and subsequently probed with fluorescent antibodies against CD9, CD63 and CD81 showing the presence of EVs that were double positive for these markers. CD9, CD63 and CD81 are EV markers, while CD41 served as a platelet marker indicating the non‐detectable contamination of blood that could have occurred during surgery. MIgG serves as an isotype control (*n* = 1, error bars = SD for the technical replicates on the chip). (d) Particle measurement of three paired mucosa and tumour isolates showed an increase of particles in relation to tissue weight for tumour tissue isolates with 11.9 × 10^10^ ± 3 × 10^10^ and 65.9 × 10^10^ ± 14 × 10^10^ particles/gram tissue for mucosa and tumour, respectively (*n* = 3, error bars = SD).

EVs could be clearly visualised by electron microscopy in samples isolated from both tumour and mucosal tissues (Figure 2a). This validates our previous findings of EVs in melanoma tumour tissue (Crescitelli et al., 2021, 2020; Jang et al., 2019). Furthermore, the background observed on the grids appeared largely free of non‐vesicular contaminants such as large protein structures, arguing for the success of the EV isolation (Karimi et al., 2018; Nordin et al., 2015). In addition, isolates analysed by Western blot were positive for the membrane proteins CD9, CD63, CD81 and Flotillin‐1, which are commonly used to demonstrate the presence of EVs in samples (Figure 2b). Additionally, Mitofilin and ADAM10, two more recently suggested markers for large and small EVs, respectively, were also detected (Kowal et al., 2016). The absence of Calnexin, a common marker for non‐EV contaminants, was also confirmed. Likewise, ExoView analysis demonstrated the presence of EVs that were double positive for any pair of the markers CD9, CD63 and CD81 and negative for CD41a, which is commonly used as a platelet marker (Figure 2c). Nanoparticle tracking analysis was performed to determine the vesicular abundance of tumour and mucosa tissue and showed that 60‐fold greater numbers of EVs could be isolated from tumour tissue than from mucosa (Figure 2d). Together, these results demonstrate that EVs can be isolated from colon cancer tissue as well as colon mucosa tissue, and this adds to our previous findings in melanoma tissue (Crescitelli et al., 2020; Jang et al., 2019).

### 3.2 Tissue‐derived EVs display common EV‐markers

EVs isolated from tumour and mucosa tissue of 10 patients were analysed in two independent TMT sets. A total of 2567 and 3742 proteins were quantified in study 1 and study 2, respectively. The differences in the two studies are likely due to optimised MS parameter in the LCMS analysis to improve quantification in study 2 as well as LCMS performance. Advantages of multiplex TMT studies is the low technical variability and reduced number of missing values in a TMT set. Moreover, the negative effect of possible patient dependent lipid co‐isolation during exosome isolation on LCMS‐analysis and quantification is minimised. Quantification reproducibility is less sensitive to performance variations of the LCMS systems than for label free quantification (LFQ) measurements. Furthermore, multiplexing allows for more feasible off‐line fractionation prior to LCMS analysis for in‐depth proteome coverage.

**FIGURE 3:**
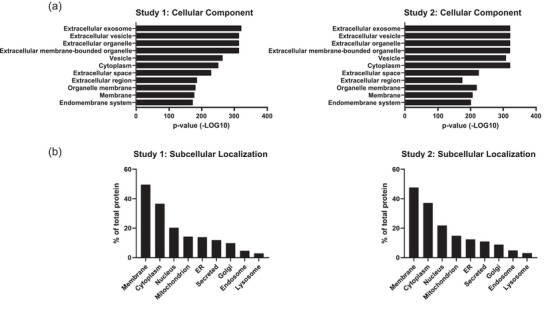
Proteomic overview of tissue‐derived EVs. (a) The top GO terms for cellular components in terms of *p*‐value that are associated with the proteins identified in the two sets. The order is according to Study 1, starting with the lowest *p*‐value on top. (b) The cellular localization of the proteins identified in the two sets. Added together, these exceed the total amount of proteins found because a protein can belong to several localization groups.

Common EV markers such as CD63, TSG101 and Flotillin 1, among others, were identified in both sets. Likewise, other relevant proteins such as components of the ESCRT machinery as well as a number of RAB proteins were among the identified proteins (Table 2). Additionally, subcellular localisations of proteins showed a predominant distribution in the membrane, which supports our previous finding that the application of a density gradient during isolation enriches for EV‐associated proteins and is crucial for proteomic studies of tissue‐derived EVs (Figure 3b) (Crescitelli et al., 2020). To further confirm similarities in the results from Study 1 and 2, gene ontology analysis of the quantified proteins was performed. As expected both studies shared overall similarity with the top 10 GO terms (Figure 3a). Overall, the two studies were comparable and were therefore analysed together in the downstream analysis.

**TABLE 2:** Identification of common EV proteins.

	Protein	Study 1	Study 2
Vesicle markers	CD9		X
	CD63	X	X
	CD81	X	X
	Flotillin 1	X	X
	Alix	X	X
	TSG101	X	X
	Annexin A5	X	X
	ADAM10	X	X
	Mitofilin	X	X
ESCRT proteins	CHMP1A,B	X	X
	CHMP2A,B	X	X
	CHMP3	X	X
	CHMP4A		X
	CHMP4B	X	X
	CHMP5	X	X
	CHMP6	X	X
	VPS4A,B	X	X
	VPS25		X
	HGS	X	X
RABs	RAB7A	X	X
	RAB11B	X	X
	RAB27A	X	X
	RAB27B		X
	RAB2A	X	X
	RAB2B		X
	RAB5A,B,C	X	X
	RAB11B	X	X
	RAB35	X	X

Proteins commonly referred to as EV markers as well as other potentially related proteins that are commonly associated with EVs and their biogenesis. An ‘X’ shows that they were successfully identified in the dataset.

### 3.3 The proteome is altered in EVs released by colon cancer tissue compared to mucosa tissue

**FIGURE 4:**
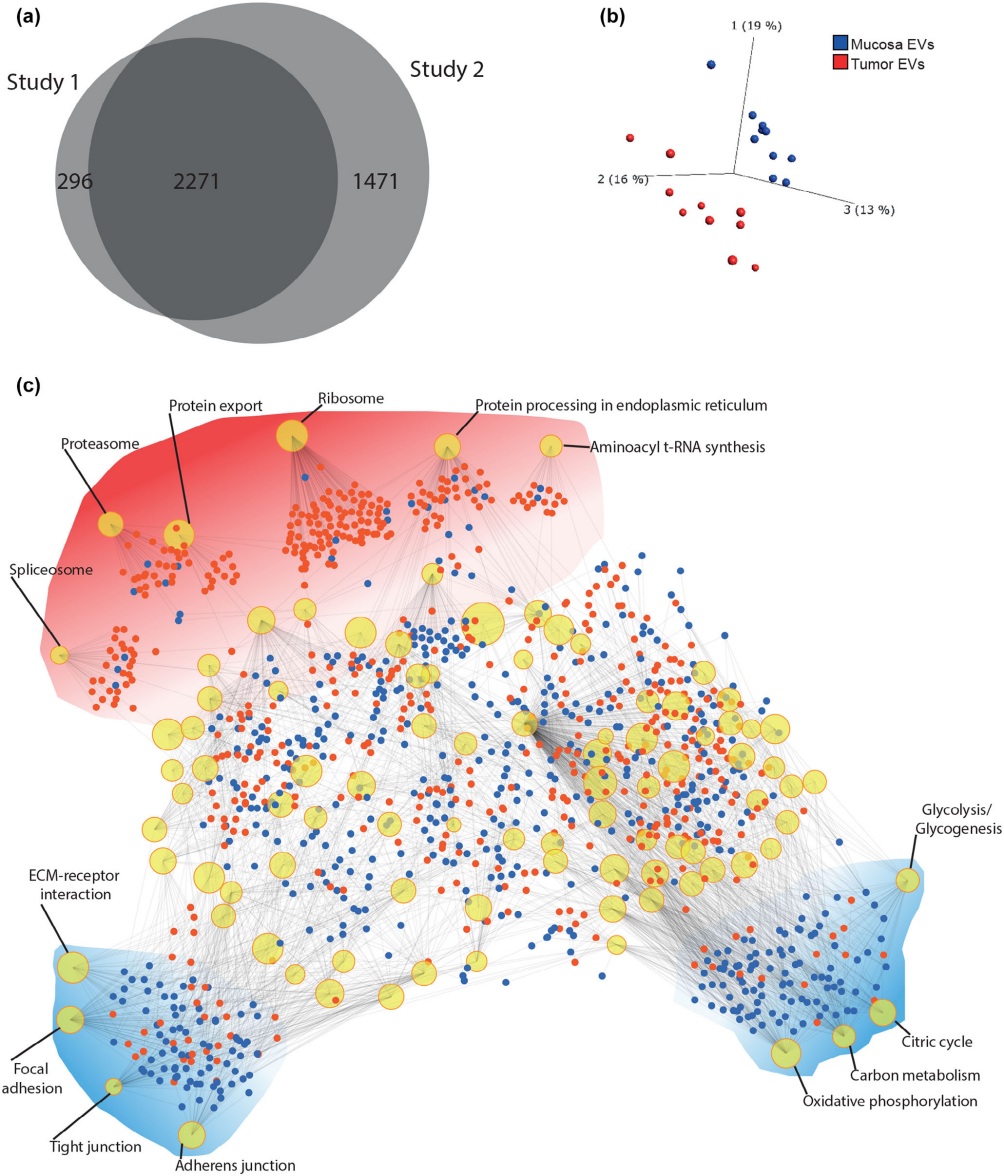
PCA and protein‐protein interactions of datasets. (a) Venn diagram of Study 1 and Study 2 showing the overlap of the two sets. (b) PCA plot constructed using the proteins that overlap in the Venn diagram. The first three components are shown with mucosa and tumour‐derived EVs in blue and red, respectively. (c) Enrichment network with the overlapping proteins as input. Nodes represent KEGG pathways. The size of the nodes corresponds to the number of genes in that particular pathway that were identified in the input list. Proteins are represented by either blue (downregulated) or red (upregulated) dots, and edges indicate to which pathway they belong. Three clusters were pulled out from the body of the network and accentuated by red (generally upregulated) or blue (generally downregulated) clouds.

To visualise the relationship among the patient samples, a PCA was performed using the proteins present in both sets. Components 1, 2, and 3 represented 48% of the overall variability in the data. A separation in the three‐dimensional plot illustrated that tumour and mucosa‐derived EVs formed distinct clusters, illustrating that much of the dissimilarities lay in tissue type rather than among patients (Figure 4b). An enrichment analysis network was generated using the commonly identified proteins in order to observe the protruding pathways (Figure 4c). Certain nodes containing largely upregulated or downregulated proteins were pulled out of the network in order to more clearly visualise the protruding pathways. This resulted in three larger clusters, of which two were skewed towards downregulation in tumour‐derived EVs as compared to mucosa‐derived EVs. These clusters contained proteins related to adhesion or involved in energy production. Specifically, among the downregulated clusters in tumour‐derived EVs were nodes representing ‘Glycolysis/Glycogenesis’ and different aspects of ATP production related to the mitochondria, as well as proteins involved in extracellular matrix interaction and junction proteins. The third cluster was skewed towards upregulation and was related to protein production. In the upregulated cluster, nodes representing protein production and degradation such as ‘Spliceosome’, ‘Ribosome’, and ‘Proteasome’ were identified among others. Taken together, the protrusions of these pathways suggest the cells to be in a state of low energy production but high protein turnover and loosely adhered to each other, if indeed the EVs are a representation of cellular phenotype.

### 3.4 Potential biomarker candidates exist within the dataset

**FIGURE 5:**
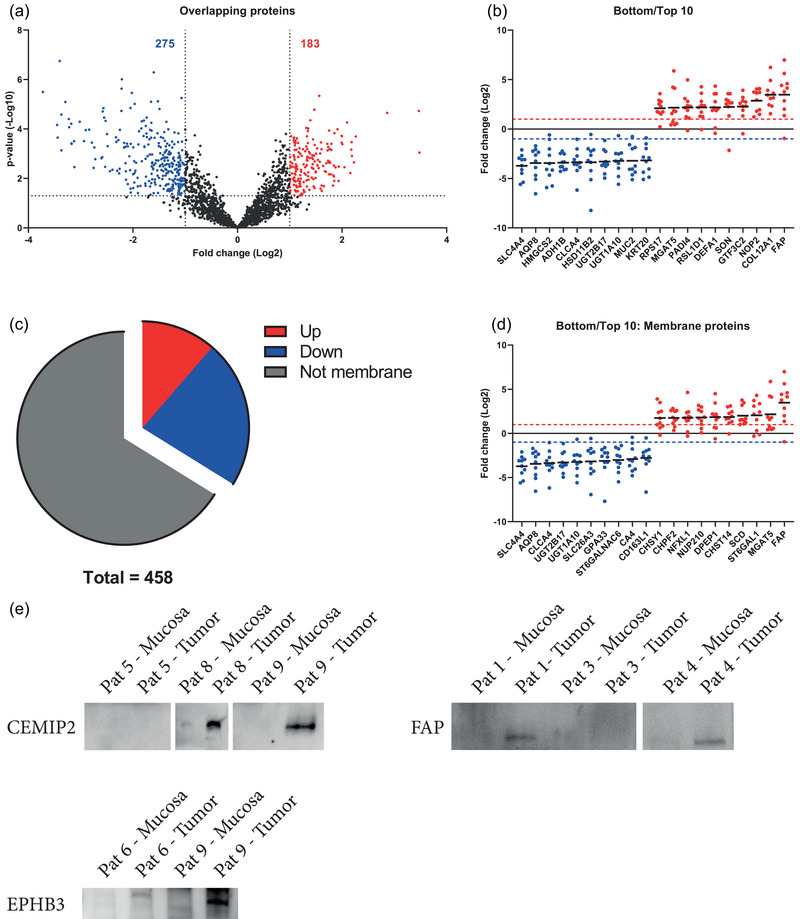
Quantitative differences in proteins between tumours tissue‐derived EVs and mucosa‐derived EVs. (a) Volcano plots of the proteins common for both sets. The dotted lines indicate the cut off, which is 1.3 on the Y‐axis (corresponding to a *p*‐value of 0.05) and 1 on the X‐axis (corresponding to a fold change of 2). Red is representative of an enrichment in tumour‐derived EVs while blue represents a decrease. (b) The top 10 upregulated and top 10 downregulated proteins in each study according to fold change (red and blue dotted lines intercept the y‐axis at a fold change of 2). (c) Pie chart showing the portion of significantly regulated proteins that are membrane proteins. (d) The top 10 upregulated and top 10 downregulated membrane proteins in tumour tissue EVs.

A cut off was set to generate a list of high‐confidence proteins. Apart from being identified in both sets, a protein needed a fold change of at least 2 and a *p*‐value of 0.05 or lower to be confidently considered as differently loaded in tumour‐derived EVs compared to mucosa‐derived EVs. In total, 183 proteins were upregulated in tumour‐derived EVs and 275 were downregulated (Figure 5a). The top 10 upregulated and downregulated proteins and the individual fold change in each patient are exemplified in Figure 5b, showing proteins such as prolyl endopeptidase FAP (FAP) and electrogenic sodium bicarbonate cotransporter 1 (SLC4A4) to be dysregulated in tumour‐derived EVs.

Special consideration was given to membrane proteins putatively displayed on the vesicular surface for two particular reasons. Their membrane association further strengthens the claim that they are indeed vesicle‐bound elements, and their particular position on the EVs makes them attractive from a biomarker perspective because they could potentially be accessible in biofluids through affinity‐based isolation methods. Subcellular localisations were acquired from Uniprot, which revealed 52 upregulated and 103 downregulated membrane proteins that also met the criteria described above (Figure 5c). The top 10 upregulated and downregulated membrane proteins are shown in Figure 5d. Three upregulated membrane proteins, FAP, CEMIP2, and Ephb3, were selected for validation with western blot based on their suitability as potential future biomarkers. The criteria for their selection included that they be up‐regulated, that they canonically localise to the plasma membrane, and that they display a large portion of the protein on the extracellular side. Western blots of these proteins showed band intensities that reflected the relative abundance as measured by mass spectrometry (Figure S1).

## 4 DISCUSSION

The primary tumour tissue interstitium represents ‘ground zero’ for the cancer cell secretome, and this is the milieu where the highest concentration of tumour signals are likely to be detectable, including that of EVs. Through a rigorous method of isolating EVs from tissues (Crescitelli et al., 2021), (Figure 1), we are able to detect relevant vesicle proteomic profiles, and we thereby avoid the presence of contaminating factors such as different soluble proteins including those that are highly abundant in blood plasma (Ishiguro et al., 2019; Jang et al., 2019; Ji et al., 2021; Sun et al., 2021; Vella et al., 2017). Using this approach, we have here compared the EV proteome profiles of primary colon cancer tissue versus non‐tumour colon mucosa. Firstly, the isolates from the tissues seem highly enriched in EVs as confirmed by vesicle marker identification, as well as gene ontology analysis. The tumour EVs were in many ways distinct from EVs isolated from non‐tumour tissues, shown by principal component analysis and enrichment analysis. A total of 275 proteins were found to be downregulated in the tumour tissue versus mucosa derived EVs, and 183 that are upregulated. Further, multiple upregulated EV membrane proteins were identified that could be possible to identify in different body fluids.

In the present study, we confirmed the presence of EVs in the isolates by several methods including TEM, western blotting, and Interferometric analysis and CD‐marker identification analysis through the ExoView platform, as well as particle tracking analysis (Figure 2). The results suggest that the EV isolates are clean, as shown by electron microscopy imaging. Further, the protein analysis identifies typical EV proteins such as the tetraspanins CD81, CD63, CD9. These are known to be present in EVs from most sources (Kowal et al., 2016), in parallel with the absence of, for example, the ER marker Calnexin, which argues that the isolates lack cellular organelle contaminations. Further, the marker identification with the ExoView platform identified the presence of multiple tetraspanins within a single‐vesicle resolution, further supporting the conclusion that the isolates contain naturally released EVs. Furthermore, the absence of the platelet marker CD41a argues that the isolated EVs are not of blood origin. Notably, particle counting showed that tumour tissues contains higher levels of EVs as compared to healthy mucosal tissue, as has been previously postulated (Whiteside, 2016). This increases the chance for their detection in circulation as their levels could be elevated sufficiently to reach a detectable signal over that of non‐tumour EVs (Jang et al., 2019; Logozzi et al., 2009; Riches et al., 2014)

To characterise the EVs from tissues, and to determine differences in protein expression between tumour and non‐tumour tissue EVs, a global quantitative proteomic study was performed. The results from the proteomic study support the concept that the EV‐isolates represent naturally released EVs, as the gene ontology analysis results in the highest term being ‘Extracellular exosome’ (Figure 3). Even though the two studies differed in the number of identified proteins, their similarity in overall protein makeup was supported by their similarities in both gene ontology and by protein subcellular localisation analysis, again supporting the conclusion that the isolates are representative EV isolates. Altogether, our results support the conclusion that the method is reproducible not only from a technical standpoint, but also from a biological one. This is further highlighted by the large number of overlapping proteins (2271 proteins in total) that were identified in both of the TMT sets (Figure 4).

Of all the proteome unveiled in this study, the overlapping proteins, which were reliably identified over two data sets and in all 10 patients, were viewed as fixed components of the tumour and mucosa microenvironment. A principle component analysis performed on these shared proteins reveals a clear clustering of tumour derived EVs away from samples of the healthy mucosa (Figure 4b), further supporting the reproducibility of the data, both from a technological and biological viewpoint. This data also strongly indicates that EVs from the tumour microenvironment carries different protein cargo than those of the normal mucosa, which may be helpful from the perspective of biomarker discovery. To evaluate what these differences might be, we performed an enrichment analysis on these shared proteins which revealed a clear downregulation of energy production machinery coupled with an equally stark upregulation of protein production machinery (Figure 4c). This supports the presence of signs of the ‘Warburg effect’ in the colon cancer EV, in which cancer cells can downregulate oxidative phosphorylation in favour of protein synthesis (Liberti & Locasale, 2016). Observing this characteristic in the tissue EVs could be a reflection of the phenotype of the cells which produced them. Interestingly, proteins related to focal adhesion, tight junctions and extracellular matrix interactions were also downregulated in tumour EVs. This is again interesting, especially if this signature is a reflection of the producing cell phenotype, as these components reflect a more migratory cell phenotype (Wu et al., 2021). In a vesicular context, this could suggest that the tissue EVs may migrate easier through the tumour microenvironment, potentially increasing their ability to reach the systemic circulation.

The proteome was further narrowed down in order to generate a list of proteins with the highest confidence of dysregulation. We found that 275 proteins were significantly downregulated in tumour‐derived EVs as compared to those of the mucosa, while 183 were found to be upregulated (Figure 5a). These shared and significantly dysregulated proteins could serve as a foundation for a pool of potential EV biomarker candidates, especially the most highly dysregulated ones that putatively reside in the membrane surface, as they potentially could be more easily accessible for antibody detection (Figure 5b–d). Three proteins were chosen for validation through western blotting, further authenticating the mass spectrometry findings (Figure S1), which can help identify EV‐based biomarker candidates (Xiao et al., 2020). Some of the most downregulated proteins in tumour‐derived EVs (Figure 5b, Table S1) may represent a de‐differentiation of epithelial cells through the loss of mucosal epithelia‐typical proteins such as transporters and mucins. For example, downregulation of the sodium bicarbonate cotransporter SLC4A4 has been shown to be coupled to the de‐differentiation of colon epithelia in cancer (Yang et al., 2020), as is also the case for mucin MUC2 (Wang et al., 2017).

Several of the proteins that were significantly upregulated in EVs from tumour tissue versus mucosal tissue have been previously associated with different cancers (Figure 5b, Table S1). For example, overexpression of FAP has been linked to negative prognosis in colon cancer (Liu et al., 2015). Similarly, Dipeptidase 1 is a protein found to be overexpressed in cancer tissue in the colon in the early stages of carcinogenesis as compared to mucosal tissue and has been suggested as a candidate marker for colorectal cancer (Eisenach et al., 2013; Toiyama et al., 2011). In vitro studies also suggest that knockdown of Dipeptidase 1 attenuates the invasiveness of the HCT116 and SW480 cancer cell lines (Hao et al., 2017). The cell surface hyaluronidase CEMIP2, a relatively newly characterised membrane‐bound hyaluronidase, is emerging as a key player in our understanding of cellular hyaluronan catabolism and has been shown to play a role in cell migration (Irie et al., 2021). These three proteins all remodel extracellular matrix components, and their circulation in the tumour microenvironment might facilitate the escape of cancerous cells from their confinement in the primary tumour (Kai et al., 2019). Another protein found to be upregulated in our study, the metallo‐endopeptidase Neprilysin (also known as CD10), has been shown to correlate with tumour weight in mouse models and higher serum concentrations were found in colorectal cancer patients with liver metastases (Sasaki et al., 2014). Its presence in EVs has been proposed through its interaction with the EV marker CD9 (Mazurov et al., 2013). Neprilysin facilitates the cleavage of a wide range of substrates, including signalling peptides, and it has multifaceted roles in different cancers as well as in different tissues (Maguer‐Satta et al., 2011). Interestingly, the cell adhesion molecule Carcinoembryonic antigen (CEA), an established biomarker for colon cancer, was also found to be upregulated in tumour‐derived EVs. Its cellular function in a cancer scenario would be that of immune evasion by interacting with the natural killer cell inhibitory receptor CEACAM1, an interaction that if blocked leads to enhanced NK cell cytotoxicity (Zheng et al., 2011). CEA has been previously shown to be secreted in EVs (Xiao et al., 2020; Yokoyama et al., 2017), and by having immune suppressive molecules such as CEA unconfined to the cellular plasma membrane, one can postulate that the vesicle‐bound form potentially further suppresses NK cell activity. These examples of proteins that were previously thought to be confined exclusively to the cells, are suggested to exist in the interstitium as parts of secreted EVs. Thus, the EVs in tumours, as well as other tissues, may be contributing to a tissue cellular interactome, specifically between cells of different types. These above‐mentioned example‐proteins amongst others have, despite the homogeneity of patient TNM status (Table 1), remained similarly regulated throughout the evaluated samples, indicating that such dysregulations are a more general feature of colorectal cancer tumours.

Based on the present study, further efforts should be spent on identifying whether or not the proteins deemed significantly dysregulated are identifiable in samples collected through less invasive methods, such as blood or faeces. This would strengthen their usefulness as potential biomarkers for disease. As would a larger study cohort, which would allow for the discovery of TNM status related protein dysregulation as well as further validate the discoveries found in this study. Furthermore, complementary proteomics on the cells of the tissue would benefit future studies of this nature as it would lend a context against which to interpret the EV proteome from a pathological viewpoint.

## 5 CONCLUSION

This study provides a snapshot and comparison of the vesicular secretomes in both colon tumour and non‐tumour tissue interstitium. EVs secreted in the tumour microenvironment differ greatly in their protein cargo vs those present in non‐tumour mucosa. This could hold clues pertaining to the phenotype of the tumour as well as the cellular processes that take place in its tumour microenvironment and beyond. Such insight is not only important in the context of understanding disease progression, but also serves as a source of potential EV‐biomarkers identification.

## AUTHOR CONTRIBUTIONS

**Aleksander Cvjetkovic**: Conceptualization; data curation; formal analysis; investigation; methodology; project administration; validation; visualization; writing—original draft; writing—review and editing. **Nasibeh Karimi**: Investigation; methodology; validation; writing—original draft; writing—review and editing. **Rossella Crescitelli**: Investigation; methodology; validation; writing—original draft; writing—review and editing. **Annika Thorsell**: Data curation; formal analysis; investigation; methodology; resources; writing—original draft; writing—review and editing. **Helena Taflin**: Methodology; resources; writing—original draft; writing—review and editing. **Cecilia Lässer**: Conceptualization; data curation; formal analysis; investigation; methodology; project administration; supervision; validation; visualization; writing—original draft; writing—review and editing. **Jan Lötvall**: Conceptualization; funding acquisition; project administration; supervision; writing—original draft; writing—review and editing.

## CONFLICT OF INTEREST STATEMENT

The authors J.L., R.C., C.L. and A.C. have developed multiple EV‐associated patents for putative clinical utilisation. J.L. owns equity in Codiak BioSciences Inc. and Exocure Biosciences Inc. and consults in the field of EVs through Vesiclebio AB. R.C., A.C. and C.L. own equity in Exocure Bioscience Inc.

## Supporting information


**Supplementary Figure 1** Western blot validation of select proteins from proteomics.

**Supplementary Table 1** Proteomics Results

## Data Availability

PX Partial: The mass spectrometry proteomics data have been deposited to the ProteomeXchange Consortium (http://proteomecentral.proteomexchange.org) via the PRIDE partner repository (Perez‐Riverol et al., 2019) with the dataset identifier PXD033236. Access for reviewers: **Username**: reviewer_pxd033236@ebi.ac.uk. **Password**: IQ8pGNUK
