# Supplementary figures and images for: Proteomic profiling of tumour tissue‐derived extracellular vesicles in colon cancer
Source: J Extracell Biol. 2024 Feb 6;3(2):e127. doi: 10.1002/jex2.127 (PMC11080707; doi:10.1002/jex2.127)

Supplementary Figure 1  
Western blot validation of select proteins from proteomics.

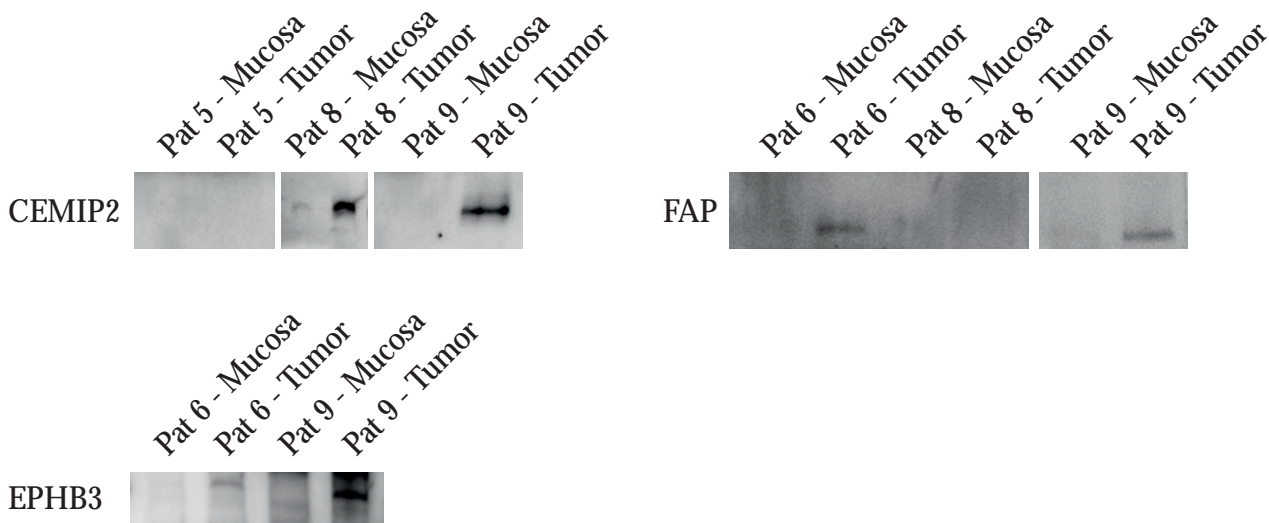

Supplement: Supplementary file 1 — Supplementary Figure 1 Western blot validation of select proteins from proteomics. [file JEX2-3-e127-s002.pdf]
